# miR-548b-3p Regulates Proliferation, Apoptosis, and Mitochondrial Function by Targeting CIP2A in Hepatocellular Carcinoma

**DOI:** 10.1155/2018/7385426

**Published:** 2018-12-23

**Authors:** Lin Lin, Yong Wang

**Affiliations:** Department of General Surgery, The Fourth Affiliated Hospital of China Medical University, China

## Abstract

The roles of miR-548b-3p in the progression of hepatocellular carcinoma (HCC) remain undiscovered. This study aims to explore the roles and mechanisms of miR-548b-3p in HCC. Using TCGA database, we found that miR-548b-3p expression was lower in HCC compared to the normal tissues, which was further confirmed by RT-qPCR of 20 cases of surgically resected HCC and corresponding normal tissues. miR-548b-3p mimic and inhibitor were transfected into Huh7 and SK-Hep-1 cells, respectively. MTT, colony formation, and cell cycle assays showed that miR-548b-3p mimic suppressed cell growth and G1/S cell cycle transition. In contrast, miR-548b-3p inhibitor facilitated cell growth and cell cycle transition. miR-548b-3p mimic also increased cisplatin sensitivity by upregulating apoptosis rate. JC-1 staining showed that miR-548b-3p mimic downregulated mitochondrial membrane potential, while miR-548b-3p inhibitor showed the opposite effects in SK-Hep-1 cells. Using prediction software, we found that CIP2A was on the target list of miR-548b-3p. miR-548b-3p mimic downregulated CIP2A and its downstream target protein c-Myc. Luciferase reporter assay demonstrated that CIP2A was as a direct target of miR-548b-3p. CIP2A depletion partly reduced the effect of miR-548b-3p mimic/inhibitor on c-Myc. CIP2A depletion also reduced the effect of miR-548b-3p mimic/inhibitor on proliferation. In conclusion, our data demonstrated that miR-548b-3p was downregulated in HCC. miR-548b-3p regulates proliferation, apoptosis and mitochondrial function by targeting CIP2A in HCC.

## 1. Introduction

Hepatocellular carcinoma (HCC) is one of the most common malignancies and it is the second most common cause of cancer-related death. As an indicator of cure rates, the median five-year survival rate was approximately 5-9% for HCC [1, 2]. The pathogenesis of HCC is a multistep and multistage process involving environmental factors and genetic factors [3]. Exploring the molecular mechanisms and searching for key molecules during initiation and progression of HCC are important for the development of novel treatment.

Cancerous inhibitor of protein phosphatase 2A (CIP2A) is an endogenous protein phosphatase 2A (PP2A) inhibitor and Myc stabilizer, which is reported to promote malignant cell transformation and tumor growth [4]. Generally, CIP2A was expressed at a low level in normal tissues [5–8]. CIP2A was upregulated in various human malignancies including ovarian cancer, head, and neck squamous cell carcinoma, non-small lung carcinoma, and gastric cancer, which was closely associated with poor patient prognosis [9–14].

microRNA is a family of small and endogenous noncoding RNAs, which were involved in the regulation of multiple biological processes including cell proliferation, migration, invasion, mitochondrial function, and apoptosis. Abnormal expression of miRNAs is attributed to many decreases. Many reports were indicating that miRNAs play a vital role in many liver diseases. miR-548 is a poorly conserved primate-specific miRNA gene family. miR-548 family has been demonstrated to be involved in the pathogenesis of several cancers. miR-548b-3p was significantly downregulated in breast cancer and regulated proliferation and apoptosis by targeting ECHS1 [15]. It is also reported that miR-548b-3p inhibits the proliferation and invasion of malignant gliomas by targeting MTA2 [16]. However, expression and biological effect of miR-548b-3p in HCC remain unclear.

In this study, we examined miR-548b-3p expression pattern in HCC. The biological effects of miR-548b-3p and its potential mechanism were also explored.

## 2. Materials and Methods

### 2.1. Patients and Tissue Specimens

This study was approved by the Ethical Committee of the Fourth Affiliated Hospital of China Medical University. Twenty HCC specimens and corresponding normal liver tissues were obtained from patients who were diagnosed with HCC and undertook surgeries in the Fourth Affiliated Hospital of China Medical University between May 2012 and February 2016. Clinical data was obtained from medical record of patients.

### 2.2. Cell Culture and Transfection

LO2, SK-Hep-1, BEL7402, and Huh7 cells were purchased from the Cell Bank of Type Culture Collection of Chinese Academy of Science. LO2, SK-Hep-1, and Huh7 cells were cultured in Dulbecco's Modified Eagle Medium (DMEM; Gibco; Thermo Fisher Scientific; USA) supplemented with 10% fetal bovine serum (FBS; Gibco; Thermo Fisher Scientific; USA). BEL7402 cells were cultured in RPMI-1640 medium (Gibco; Thermo Fisher Scientific; USA) with 10% FBS. These cells were cultured in 37°C incubator with saturated humidity and 5% CO_2_.

Cells in the logarithmic growth phase were inoculated into 6-well culture plates and used for transfection. CIP2A siRNA and nontargeting siRNA were transfected using DharmaFECT 1 reagent according to the manufacturer's instruction. MiR-548b-3p mimic, mimic control, miR-548b-3p inhibitor, and inhibitor control were purchased from RiboBio (Guangzhou, China). DharmaFECT 1 (Dharmacon, Lafayette, CO, USA) was used for miR mimic and inhibitor transfection. For induction of cell apoptosis, CDDP was applied to the transfected cells for 24 hours.

### 2.3. Western Blot

Cell lysates were prepared using lysis buffer with protease and phosphatase inhibitor. Total protein was extracted and quantified. Sample protein (40 *μ*g) was resolved through SDS-PAGE and transferred to polyvinylidene fluoride (PVDF) membranes. Then PVDF membranes were blocked with 5% BSA for 1 hour at room temperature. Primary antibodies CIP2A c-Myc, cleaved PARP, and actin (1:1000, Cell Signal Technology, USA) were incubated at 4°C overnight. PVDF membranes were rinsed with TTBS buffer and incubated with secondary anti-mouse/rabbit IgG HRP-linked antibody (1:2000, Cell Signal Technology, USA) at 37°C for 1 hour. Proteins were visualized by ECL kit (Thermo Fisher Scientific; USA) in a DNR Bio-Imaging system (DNR Bio-Imaging Systems, Israel).

### 2.4. Real-Time Fluorescent Quantitative PCR

Total RNA was extracted from tissue and cells using Trizol reagent (Sigma Aldrich, USA). Total RNA served as a template, and then cDNA was synthesized using PrimeScript RT Master Mix (Takara, Dalian, China)/PrimeScript RT reagent kit (Takara, Dalian, China) according to manufacturer's instructions.

cDNA amplification was performed according to SYBR Green PCR Master Mix (Applied Biosystems; Thermo Fisher Scientific, USA) kit using ABI 7500 Real-Time PCR system (Applied Biosystems; Thermo Fisher Scientific, USA). *β*-actin was used as the reference gene of CIP2A amplification, and U6 was used as the reference gene of miR-548b-3p sequence amplification. The amplification multiples were calculated through 2^−ΔΔCT^ method.

### 2.5. MTT Assay

Cell viability was determined using MTT assay. The cells were cultured for 1, 2, 3, 4, and 5 days, respectively, and then treated with 20*μ*l MTT solution (5 mg/ml) for another 4 hours. 3-(4,5-Dimethylthiazol-2-yl)-2,5-di-phenyltetrazolium bromide was applied to the cells. The MTT formazan was dissolved in DMSO. OD value at 450 nm was measured using a microplate reader. The experiment was repeated in triplicate.

### 2.6. Mitochondrial Membrane Potential Assay

Briefly, cells were harvested, washed with PBS, and incubated with 5 *μ*M JC-1 (Cell Signaling Technology) for 30 minutes in the incubator. The changes of mitochondrial membrane potential were detected and analyzed by the flow cytometer (ACEA, USA).

### 2.7. Cell Cycle and Apoptosis Assay

Cells were fixed with 1% paraformaldehyde, which were stained with propidium iodide in PBS with RNase A for about 30 min. Cell cycle analysis was analyzed using flow cytometer.

Annexin V/PI staining kit from BD bioscience was used to determine the rate of apoptosis according to the manufacturer's protocol. Briefly, cells were digested and centrifuged at low-speed of 1500rpm for 10min. Then the cells were washed with precooled PBS buffer. Annexin V-FITC and PI were incubated with cell suspension. Apoptosis rate was detected and analyzed using flow cytometer.

### 2.8. Luciferase Reporter Assay

Huh7 cells were seeded in 24-well plates till the confluence reached 60%. MiR-548b-3p mimics, luciferase reporter, and pRL-TK vector carrying CIP2A 3′-UTR (binding sites: CUGAUCU) or CIP2A mutated 3′-UTR (binding sites: CGGGCGU) were cotransfected into the cells. The transfected cells were cultured for another 24 hours in an incubator and then fluorescence intensity was detected. The independent experiments were performed in triplicate.

### 2.9. Bioinformatic Analysis

miR-548b-3p expression in 39 HCC tissues and paired adjacent normal tissues from TCGA (https://cancergenome.nih.gov/) were analyzed.

### 2.10. Statistical Analysis

Windows SPSS18.0 (SPSS, Inc., Chicago, IL, USA) was used for statistical analysis. Data were presented as the mean ± standard error. The Student's t-test was used in different treated groups for statistical analysis.* P*<0.05 was considered to indicate a statistically significant difference.

## 3. Results

### 3.1. miR-548b-3p Expression Is Downregulated in HCC

**Figure 1:**
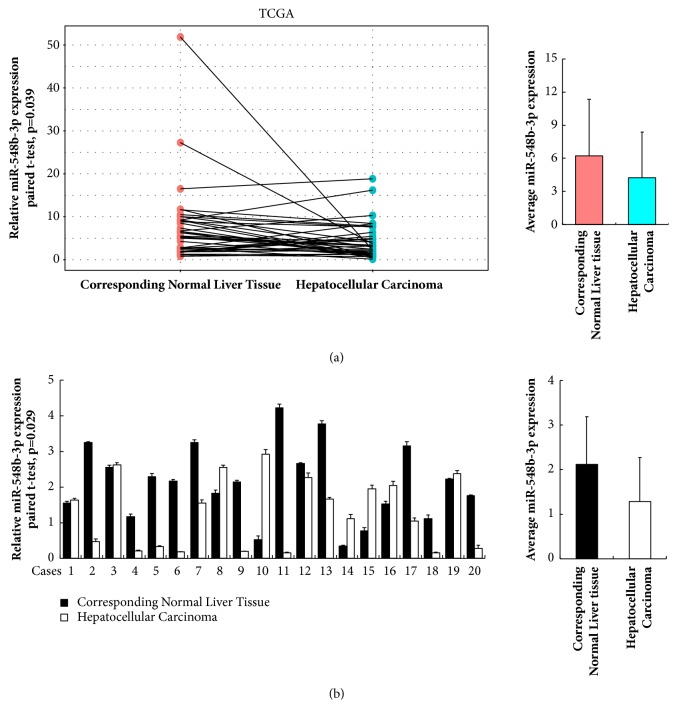
**Expression of miR-548b-3p in HCC.** (a) Analysis of RNA-seq data obtained from TCGA database showed that miR-548b-3p was lower in HCC tissues compared with corresponding normal liver tissues (n=39). (b) RT-qPCR of miR-548b-3p in 20 paired HCC/normal tissues showed that miR-548b-3p level was significantly lower in cancer tissues (n=20).

To discover the role of miR-548b-3p in the development of HCC, we first used the TCGA data for bioinformatic analysis. miR-548b-3p expression data in 39 cases of HCC together with their corresponding normal tissues was obtained and our analysis showed that miR-548b-3p was downregulated in HCC compared with paired normal liver tissues (paired Student's t-test, p=0.039, Figure 1(a)). We also examined the expression of miR-548b-3p in 20 cases of HCC tissues and their corresponding normal liver tissues using RT-qPCR. As shown in Figure 1(b), miR-548b-3p level was lower in HCC tissues compared with corresponding normal tissues (paired Student's t-test, p=0.029).

### 3.2. miR-548b-3p Inhibits Cell Proliferation and Cell Cycle Transition in HCC Cells

**Figure 2:**
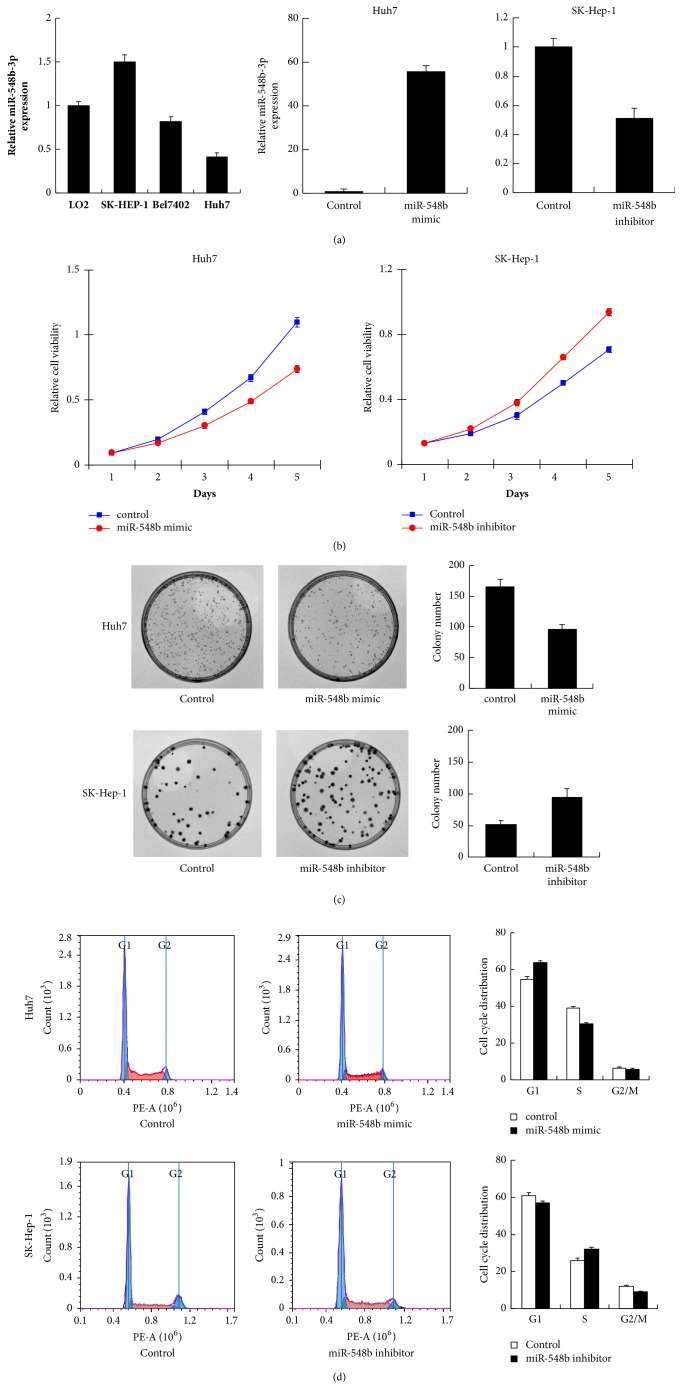
**miR-548b-3p inhibits HCC cell proliferation and cell cycle transition.** (a) Expression of miR-548b-3p in normal liver cell line LO2(HL-7702) and 3 HCC cell lines (SK-Hep-1, Bel7402, and Huh7). miR-548b-3p mimic upregulated its expression in Huh7 cell line and miR-548b-3p inhibitor suppressed its expression in SK-Hep-1 cell line. (b) MTT assay demonstrated that miR-548b-3p mimic suppressed Huh7 cell proliferation while miR-548b-3p inhibitor accelerated SK-Hep-1 cell proliferation. (c) Colony formation assay demonstrated that miR-548b-3p mimic reduced colony numbers in Huh7 cells while miR-548b-3p inhibitor upregulated colony numbers in SK-Hep-1 cells. (d) Cell cycle analysis showed that miR-548b-3p mimic upregulated G1 phase percentage and downregulated S phase percentage. In contrast, miR-548b-3p inhibitor downregulated G1 phase percentage and upregulated S phase percentage.

Next we checked the level of miR-548b-3p in normal liver cell line LO2(HL-7702) and 3 HCC cell lines (SK-Hep-1, Bel7402, and Huh7). Expression of miR-548b-3p was lower in Bel7402 and Huh7cell lines compared with LO2 (Figure 2(a)). We picked Huh7 and SK-Hep-1 cell lines for transfection of miR-548b-3p mimic and inhibitor, respectively. The transfection efficiency was determined by RT-qPCR (Figure 2(a)). miR-548b-3p mimic upregulated its expression while inhibitor suppressed its expression. MTT assay showed that miR-548b-3p mimic suppressed Huh7 cell proliferation while miR-548b-3p inhibitor accelerated SK-Hep-1 cell proliferation (Figure 2(b)). Using colony formation assay, we demonstrated that miR-548b-3p mimic reduced colony formation ability in Huh7 cells while its inhibitor upregulated colony formation ability in SK-Hep-1 cells (Figure 2(c)).

Using cell cycle analysis, we found that miR-548b-3p mimic increased G1 phase percentage and decreased S phase percentage. Treatment with miR-548b-3p inhibitor decreased G1 phase percentage and increased S phase percentage, thus facilitating G1/S progression. These results indicated that miR-548b-3p inhibited proliferation and induced G1/S arrest.

### 3.3. miR-548b-3p Upregulated Cisplatin Sensitivity and Regulated Mitochondrial Membrane Potential

**Figure 3:**
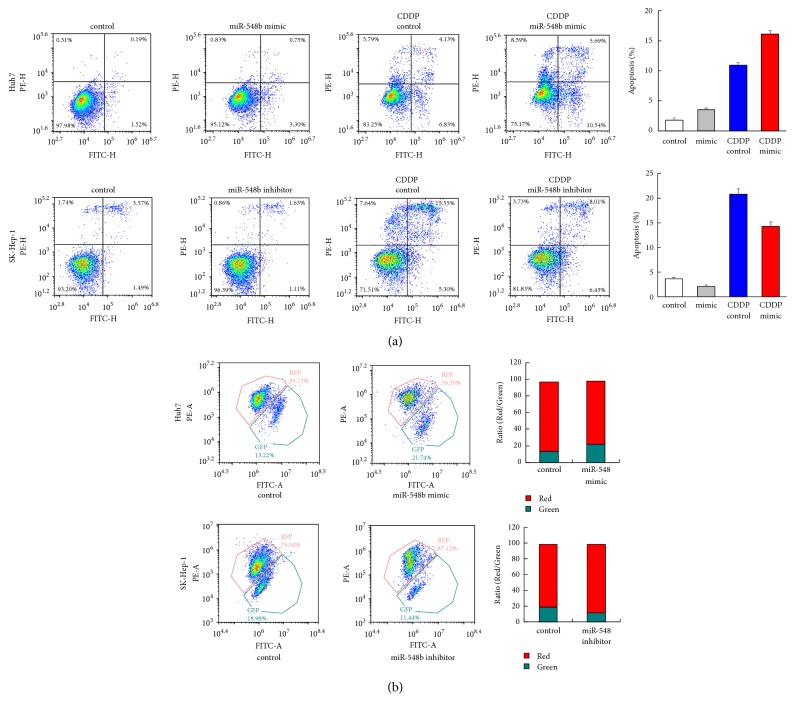
**miR-548b-3p regulates cisplatin sensitivity and mitochondrial membrane potential.** (a) In Huh7 cells treated with cisplatin, miR-548b-3p mimic increased the rate of apoptosis. In contrast, miR-548b-3p inhibitor downregulated CDDP-induced apoptosis rate in SK-Hep-1 cells. (b) JC-1 staining showed that miR-548b-3p mimic upregulated the percentage of green fluorescence, which indicated MMP decrease. In contrast, miR-548b-3p inhibitor downregulated the percentage of green fluorescence.

We then examined the effect of miR-548b-3p on cisplatin-induced apoptosis and mitochondrial function in HCC. Annexin V/PI staining was performed in HCC cells after CDDP treatment (10uM). Our results indicated that miR-548b-3p mimic increased CDDP-induced apoptosis rate in Huh7 cells (Figure 3(a)). miR-548b-3p inhibitor decreased CDDP-induced apoptosis rate in SK-Hep-1 cells.

Sensitivity to chemotherapeutic drugs is closely related to mitochondrial function; we examined whether miR-548b-3p influences mitochondrial membrane potential (MMP) in HCC cells. JC-1 staining was used to monitor the change of mitochondria membrane potential after CDDP treatment. JC-1 staining exhibits red fluorescence under normal condition while it turns into green fluorescence when MMP is downregulated after CDDP treatment. As shown in Figure 3(b), miR-548b-3p mimic decreased mitochondrial membrane potential while miR-548b-3p inhibitor increased mitochondrial membrane potential in HCC cells.

### 3.4. miR-548b-3p Targets and Downregulates CIP2A in HCC Cells

**Figure 4:**
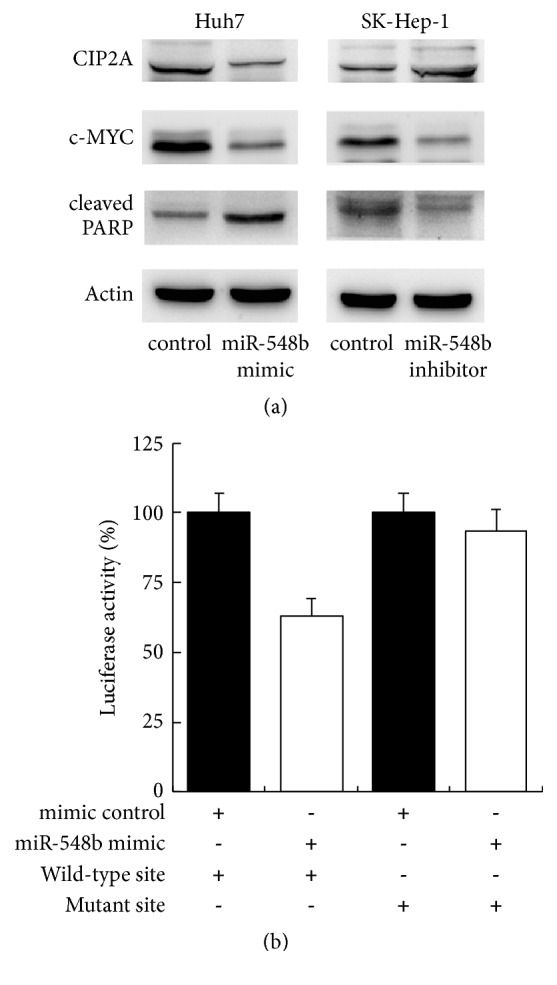
**miR-548b-3p targets and downregulates CIP2A.** (a) Western blot demonstrated that miR-548b-3p mimic decreased CIP2A and c-myc expression while upregulating cleaved PARP in Huh7 cells. miR-548b-3p inhibitor showed the opposite effect in SK-Hep-1 cells. (b) miR-548b-3p mimic suppressed the luciferase activity of wild-type reporter, while no significant change was observed in that of the mutant reporter.

**Figure 5:**
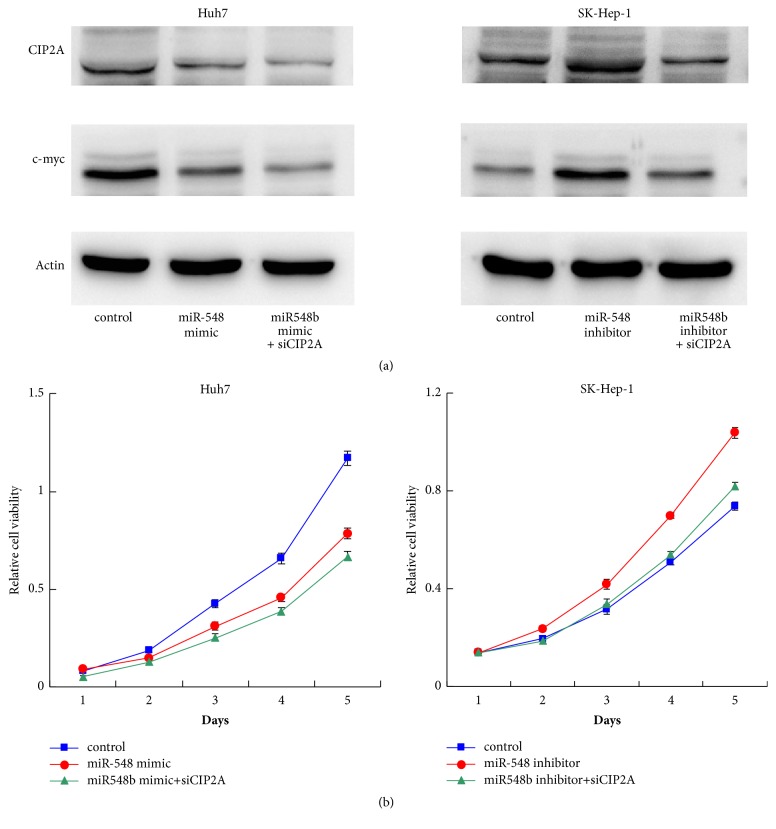
**miR-548b-3p regulates proliferation via CIP2A.** (a) Western blot demonstrated that CIP2A siRNA depletion downregulated c-myc expression in SK-Hep-1 cells treated with miR-548b-3p inhibitor. The effect of CIP2A siRNA on c-myc was not significant in Huh7 cells treated with miR-548b-3p mimic. (b) MTT assay showed that CIP2A depletion could abolish the effect of miR-548b-3p inhibitor on proliferation in SK-Hep-1 cells. The effect of CIP2A siRNA was not significant in Huh7 cells treated with miR-548b-3p mimic.

We then predicted the downstream targets of miR-548b-3p and we found that CIP2A, an important oncogene, was on the targets list. We further examined the effect of miR-548b-3p on CIP2A expression. Western blot showed that transfection of miR-548b-3p mimic significantly decreased CIP2A protein expression. In accordance, miR-548b-3p inhibitor increased expression of CIP2A (Figure 4(a)). We also checked c-myc expression, which is a downstream target of CIP2A. As shown in Figure 5(a), miR-548b-3p mimic downregulated c-myc while miR-548b-3p inhibitor upregulated c-myc expression (Figure 4(a)). miR-548b-3p also inhibited cleavage of PARP. To validate if CIP2A is the direct target of miR-548b-3p, reporters with wild-type (CUGAUCU) and mutant (CGGGCGU) 3′-UTR binding sites of CIP2A were introduced into Huh7 cells together with miR-548b-3p mimic. Luciferase reporter assay showed that miR-548b-3p mimic suppressed the luciferase activity of wild-type reporter, while no significant change was observed in that of the mutant reporter (Figure 4(b)). These results indicated that miR-548b-3p regulates CIP2A by directly targeting its 3′UTR.

### 3.5. miR-548b-3p Regulates Proliferation via CIP2A in HCC Cells

To determine the functional significance of CIP2A in miR-548b-3p induced phenotype, we depleted CIP2A expression in HCC cells transfected with miR-548b-3p mimic or inhibitor. Western blot showed that CIP2A depletion downregulated c-Myc expression in Huh7 cells transfected with miR-548b-3p mimic (Figure 5(a)). CIP2A depletion also partly abolished the effect of miR-548b-3p inhibitor on c-Myc in SK-Hep-1 cells. In addition, MTT assay showed that CIP2A depletion could abolish the effect of miR-548b-3p inhibitor on proliferation in SK-Hep-1 cells (Figure 5(b)). The cell viability in Huh7 cells with miR-548b-3p mimic and CIP2A siRNA was slightly lower than cells with miR-548b-3p mimic. These results suggest CIP2A/c-Myc plays an important role in miR-548b-3p induced growth arrest.

## 4. Discussion

miR-548 family has been reported to be involved in tumor suppression, which was associated with tumor suppressor Fhit [17]. Recent study showed that miR-548b-3p is downregulated in glioma tissues and cell lines [16]. miR-548b-3p also functions as a tumor suppressor in human breast cancer [18]. No study has assessed the involvement of miR-548b-3p in HCC. Here, we explored the expression and biological roles of miR-548b-3p in HCC development and the potential molecular mechanism. Our results showed that miR-548b-3p expression was decreased in 20 cases of HCC tissues compared with corresponding normal tissues. These findings were further validated in a RNA-seq data cohort from the Cancer Genome Atlas (TCGA), which contains 39 cases of paired HCC tissues and normal tissues. We noticed that miR-548b-3p was higher in SK-Hep-1 cell line compared with normal LO2 cell line. Since the deviation of miR-548b-3p is quite large in HCC tissues, in certain cases, its level in HCC tissue could be higher than its corresponding normal tissue. Thus it is possible that miR-548b-3p expression in certain HCC cell line such as SK-Hep-1 is higher than normal cell line LO2. These results were in accord with previous reports indicating miR-548b-3p as a potential tumor suppressor in human cancers.

For the first time, we showed the role of miR-548b-3p in regulating HCC proliferation and CDDP-induced apoptosis. Upregulation of miR-548b-3p in HCC cells using miR mimic inhibited proliferation and increased CDDP-induced apoptosis. Chemotherapeutic drugs, including cisplatin, induce apoptosis through mitochondria-dependent pathway, which makes mitochondria function important during the development of chemoresistance. The most prominent roles of mitochondria are to produce the energy currency of the cell and to regulate cellular metabolism including signaling of mitochondrial reactive oxygen species, membrane potential, and apoptosis [19, 20]. Loss of mitochondrial membrane potential (MMP) triggers apoptosis through mitochondria-dependent pathway [21]. We found that miR-548b-3p overexpression downregulated MMP while miR-548b-3p inhibitor upregulated MMP. To our knowledge, this is the first study showing that miR-548b-3p plays a crucial role in the regulation of mitochondrial function.

Next we examined the possible mechanism of miR-548b-3p in HCC cells. We showed a mechanistic link between miR-548b-3p and CIP2A, which has been reported as an important oncogene in various human cancers. First, TargetScan software indicated that miR-548b-3p could bind to 3′-UTR of CIP2A. Second, miR-548b-3p mimic downregulated CIP2A while its inhibitor upregulated CIP2A protein expression. Third, luciferase reporter assay showed that the luciferase activity was downregulated in Huh7 cells transfected with both wild site reporter and miR-548b-3p mimic. These results together demonstrated that miR-548b-3p acts as a tumor suppressor by directly targeting CIP2A.

We also demonstrated that miR-548b-3p downregulated CIP2A downstream target protein c-Myc. The biological effects of CIP2A are largely dependent on its stabilization of c-Myc oncoprotein, which drives cancer cell proliferation by upregulating cyclins and downregulating p21. The role of c-Myc on chemoresistance of HCC has also been indicated. Oncogenic c-Myc could protect HCC cells against sorafenib-induced apoptosis [22]. These results indicated that the biological effect of miR-548b-3p might depend on CIP2A target c-Myc.

c-Myc also plays an important role in mitochondrial biogenesis and function. c-Myc has been shown to bind to the promoters of genes encoding proteins involved in mitochondrial function [23]. Downregulation of c-Myc leads to a decrease in mitochondrial mass and loss of mitochondrial fusion and mitochondrial membrane potential [24]. We demonstrated that miR-548b-3p inhibited mitochondrial membrane potential, which may be the result of downregulation of CIP2A and c-Myc.

To further verify the participation of CIP2A in the biological effects induced by miR-548b-3p, cells were cotransfected with miR-548b-3p mimic/inhibitor and CIP2A siRNA. CIP2A siRNA downregulated expression of CIP2A and c-myc induced by miR-548b-3p inhibitor. CIP2A siRNA also abolished the effect of miR-548b-3p inhibitor on proliferation. Accordingly, the role of CIP2A siRNA on proliferation was not significant in Huh7 cells treated with miR-548b-3p mimic, indicating that CIP2A is essential to the biological effect of miR-548b-3p.

## 5. Conclusions

In conclusion, miR-548b-3p was downregulated in HCC and regulates HCC cell proliferation, chemosensitivity, and mitochondrial function by targeting CIP2A/c-myc. Induction of miR-548b-3p may potentiate CDDP responsiveness in HCC.

## Data Availability

The TCGA data which was used to support this study is available at TCGA website (https://cancergenome.nih.gov/).
